# Exhaled Breath Condensate: Technical and Diagnostic Aspects

**DOI:** 10.1155/2015/435160

**Published:** 2015-05-27

**Authors:** Efstathia M. Konstantinidi, Andreas S. Lappas, Anna S. Tzortzi, Panagiotis K. Behrakis

**Affiliations:** ^1^Smoking and Lung Cancer Research Centre, Hellenic Cancer Society, 8 Doryleou Street, 11521 Athens, Greece; ^2^Biomedical Research Foundation, Academy of Athens, 4 Soranou Ephessiou Street, 11527 Athens, Greece

## Abstract

*Purpose*. The aim of this study was to evaluate the 30-year progress of research on exhaled breath condensate in a disease-based approach. *Methods*. We searched PubMed/Medline, ScienceDirect, and Google Scholar using the following keywords: exhaled breath condensate (EBC), biomarkers, pH, asthma, gastroesophageal reflux (GERD), smoking, COPD, lung cancer, NSCLC, mechanical ventilation, cystic fibrosis, pulmonary arterial hypertension (PAH), idiopathic pulmonary fibrosis, interstitial lung diseases, obstructive sleep apnea (OSA), and drugs. *Results*. We found 12600 related articles in total in Google Scholar, 1807 in ScienceDirect, and 1081 in PubMed/Medline, published from 1980 to October 2014. 228 original investigation and review articles were eligible. *Conclusions*. There is rapidly increasing number of innovative articles, covering all the areas of modern respiratory medicine and expanding EBC potential clinical applications to other fields of internal medicine. However, the majority of published papers represent the results of small-scale studies and thus current knowledge must be further evaluated in large cohorts. In regard to the potential clinical use of EBC-analysis, several limitations must be pointed out, including poor reproducibility of biomarkers and absence of large surveys towards determination of reference-normal values. In conclusion, contemporary EBC-analysis is an intriguing achievement, but still in early stage when it comes to its application in clinical practice.

## 1. Introduction

Exhaled breath condensate (EBC) analysis is a recent, noninvasive method of detecting biomarkers, mainly coming from the lower respiratory tract. EBC is collected during quiet breathing, as a product of cooling and condensation of the exhaled aerosol [1]. It is a unique technique among lung function tests in terms of identifying molecular pathways, which reflect the airway epithelial function. An additional advantage is the easy, subject-friendly sampling procedure requiring only tidal breathing, in contrast with relative invasive and technically challenging methods such as bronchoalveolar lavage (BAL) [2].

There is detailed literature focused on detection of inflammatory markers, which reflect the state of chronic airways diseases such as chronic obstructive pulmonary disease (COPD), asthma, and cystic fibrosis (CF) [1]. However, contemporary methodology focus on EBC throughout analysis, with a view to identification of metabolic [3], proteomic [4, 5], and genomic [6] fingerprints of breathing, aiming for an early diagnosis of not only respiratory [4, 6] but also systemic diseases [7–9].

There is undoubtedly an increasing trend of biomedical research towards development of noninvasive, subject-friendly respiratory function testing. Furthermore, there is rapidly increasing number of original research articles regarding potential clinical applications of EBC-analysis, using various methodologies. Therefore, we aimed to present a systematic review of the literature describing the 30-year progress of research on exhaled breath condensate in a disease-based approach, with special reference to the potential clinical applications and limitations of the contemporary EBC-analysis.

We searched the PubMed/Medline, ScienceDirect, and Google Scholar databases using the following keywords: exhaled breath condensate, EBC, biomarkers, pH, asthma, gastroesophageal reflux, GERD, chronic obstructive pulmonary disease, smoking, COPD, lung cancer, mechanical ventilation, non small lung cancer, NSCLC, cystic fibrosis, pulmonary arterial hypertension, PAH, idiopathic pulmonary fibrosis, interstitial lung diseases, obstructive sleep apnea, OSA, and drugs.

We found 12600 related articles in total in Google Scholar, 1807 in ScienceDirect, and 1081 in PubMed/Medline, published from 1980 to October 2014. We used the following as exclusion criteria: (i) studies available only in languages other than English, (ii) studies published only in the form of abstract, (iii) studies published in journals without impact factor, and (iv) studies applying EBC-analysis in veterinary medicine. Furthermore, we preferably used original investigation articles instead of review articles, when possible.

207 original investigation papers and 21 review articles where eligible after applying the abovementioned criteria. 47 of them refer to methodological and technical aspects and 181 refer to the diagnostic aspects of EBC-analysis.

## 2. Methodology of EBC Collection and Analysis

### 2.1. EBC Collection Devices

EBC collection is a simple, noninvasive technique, requiring only quiet breathing of the subject, via a system of exhaled air cooling. After applying a nose-clip, the subject breaths quietly for 10 minutes through a special mouthpiece with a salivary trap and a single-way valve attached, diverting the exhaled airflow through a Teflon or polypropylene tube inside a cooling container. There, exhaled air in the form of droplets is converted to exhaled breath condensate (EBC) [1, 10].

Various house-built devices have been described, based on two simple layouts: (a) a Teflon tube, dipped in a bucket filled with ice and (b) a double glass layer container, where exhaled air condensation takes place between the two layers [1]. Commercially available devices are EcoScreen, Turbodeccs, Rtube, and Anacon glass condenser, based on the abovementioned layouts.

**Figure 1:**
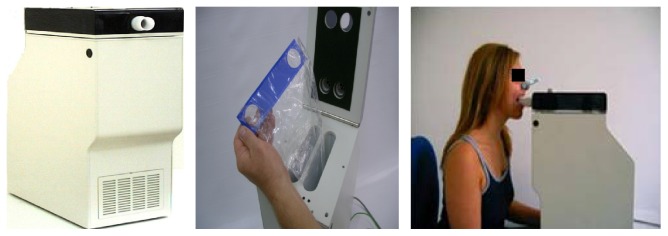
EcoScreen 2 device for exhaled breath condensate (EBC) collection (FILT Lungen-& Thorax Diagnostik GmbH). EcoScreen 2 electrically refrigerates exhaled air, conducted through a lamellar PFTE coated aluminum double lumen system. EBC is formatted in a disposable polyethylene bag (at approximately −10°C). It also allows the fractionated collection of EBC from different areas of the bronchial tree into two disposable polyethylene bags, so that the dead space condensate which contains biomarkers of no clinical relevance can be discarded. The device is not portable and weighs 20 kg (*published with permission from FILT Lungen-& Thorax Diagnostik GmbH*).

The EcoScreen device electrically refrigerates exhaled air conducted through a lamellar PFTE coated aluminum double lumen system. EBC is then formatted in a disposable polypropylene collecting cup (at approximately −10°C). EcoScreen 1 device has been extensively used in research protocols. However, it is not currently manufactured due to several technical drawbacks, including lack of manual control of condensing temperature and cleaning requirements of the device between consequent trials [11]. Therefore, the EcoScreen 1 has been replaced by EcoScreen 2 device, which allows the fractionated collection of EBC from different areas of the bronchial tree into two disposable polyethylene bags, so that the dead space condensate which contains biomarkers of no clinical relevance may be discarded. EcoScreen 1 and 2 are not portable and weight 20 kg (Figure 1).

**Figure 2:**
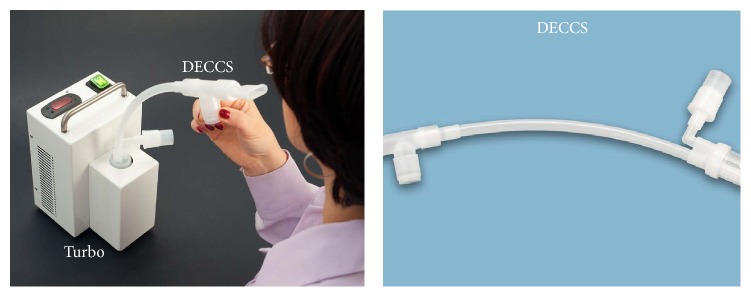
TurboDECCS device for exhaled breath condensate (EBC) collection (Medivac SRL, Italy). TurboDECCS consists of a portable Turbo Unit and a disposable DECCS collection system. DECCS is equipped with a mouthpiece, a one-way valve, a tube, and a collection cell inserted in a Peltier-type electrical cooling system* (published with permission from Medivac SRL, Italy)*.

TurboDECCS consists of a portable Turbo Unit and a disposable DECCS collection system. DECCS is equipped with a mouthpiece, a one-way valve, a tube, and a collection cell inserted in a Peltier-type electrical cooling system (Figure 2).

**Figure 3:**
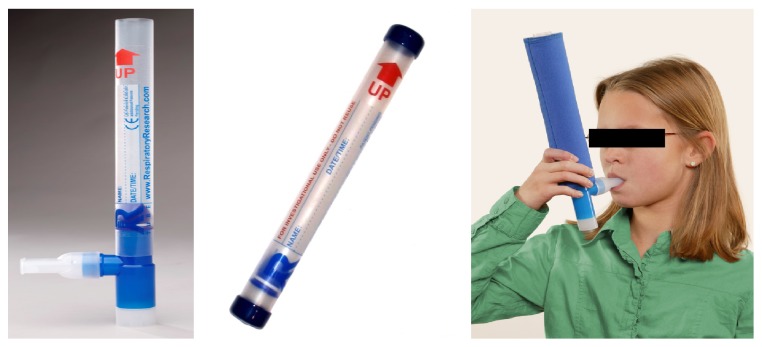
RTube device for exhaled breath condensate (EBC) collection (Respiratory Research, Inc., USA). RTube disposable collection system consists of a large Tee section (from polypropylene (PP)), which separates saliva from the exhaled breath, an one-way valve (from silicone rubber), and a PP collection tube, which is cooled by a cooling-sleeve placed around. RTube is portable and can be used by unsupervised subjects at home (*published with permission from Respiratory Research, Inc., USA*).

RTube is also portable and can be used by unsupervised subjects at home. RTube disposable collection system consists of a large Tee section (made of polypropylene (PP)), which separates saliva from the exhaled breath, a one-way valve (made of silicone rubber), and a PP collection tube, which is cooled by a cooling-sleeve placed around (Figure 3).

**Figure 4:**
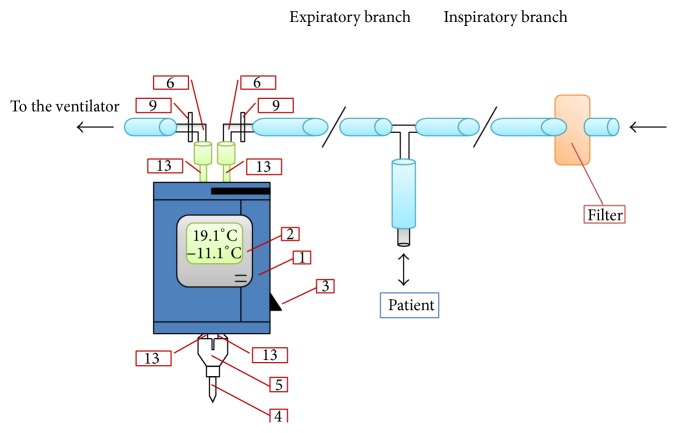
ANACON (Biostec, Valencia, Spain) condenser integrated in the mechanical ventilation circuit. The condenser is inserted in the expiratory branch of the ventilation circuit via 2 adaptors (9) and 2 elastomeric connectors (6). The exhaled air passes towards the condensation tubes (13) that pass through the body of the condenser (1). A Y piece (5) closes the circuit with the collection tube for the exhaled breath condensate (4). A thermometer (2) allows the condensation temperature to be monitored. The apparatus also contains a cooling switch (3) (*reproduced with permission from* [12]).

ANACON condenser has been used by many research groups, especially in mechanically ventilated patients [10, 12]. It is a condensing device which can be attached to the expiratory branch of the ventilator circuit. Condensation temperature is constantly monitored within the limits of −15°C to −5°C (Figure 4).

### 2.2. Sampling

#### 2.2.1. Sampling Duration

After 10 min of quiet breathing, 1–3 mL EBC is collected from adult subjects (*V*′ = 100 L/min) [1]. Sampling duration varies among studies from 10 to 30 min, but collection of up to 60 min has also been described [13, 14]. In EcoScreen device, total EBC volume is proportional with total exhaled volume, breathing frequency and total EBC protein and urea concentrations [15]. However, when using Rtube, where the temperature of cooling chamber is increasing with condensation, not much EBC is added after 10 min of sampling.

10 min collection is usually applied on adults and children over 4 years of age, as it is an easily tolerated period from the majority of subjects and provides sufficient EBC volume. During this time, released particles range between 0.1 and 4 particles × cm^3^, which have a mean diameter of 0.3 *μ*m [16].

#### 2.2.2. Sampling Conditions

There are many studies investigating the effect of environmental conditions and different breathing patterns on the volume and composition of EBC. Increased *V*′_*E*_ (ventilation per minute) resulted in significantly increased volume of EBC [17–19]. In particular, McCafferty et al. [17] demonstrated that *V*′_*E*_ of 7.5 L/min, 15 L/min, and 22.5 L/min resulted in EBC volume of 627 *μ*L, 1019 *μ*L, and 1358 *μ*L, respectively, whereas lower *V*_*T*_ (tidal volume) resulted in smaller EBC volumes. Proteins, nitrites, and pH had no change with different breathing patterns. In the same study, EBC volume was also reduced when cool and dry air was inspired. When an additional resistance of 5 cmH_2_O (Res5) was applied to the outflow tract of EcoScreen device by Davidsson et al. [20]; an increased EBC volume was collected due to the recruitment of additional alveoli.

Several studies investigated the effect of different collection conditions on certain EBC biomarkers. In a study by Vaughan et al. [14], temperature (−44°C to +13°C) and duration of collection (2–7 min), acute airway obstruction (after methacholine challenge), profound hypoventilation, and hyperventilation were proved to have no effect on EBC pH. Contrarily, in a study by Kullmann et al. [21] environmental temperature and relative humidity were found to contribute to the variability of EBC-pH and it was suggested that these factors should be controlled as part of the standardization of EBC collection. Regarding H_2_O_2_ levels in EBC, Schleiss et al. [22] demonstrated that they were flow-dependent at expiratory flow rates between 48 and 140 mL/s.

#### 2.2.3. EBC Origin

The dominant component of EBC is condensed vapour (99.9% of EBC volume). Therefore, water soluble volatile compounds (acids and bases) are highly diluted. Daily, 350 mL of water is lost through breathing and released as vapor from the airways surface [23]. Total amount of water lost is determined from ventilation per minute (*V*′_*E*_), as exhaled breath is almost completely saturated with water vapor [24].

Apart from water soluble volatile compounds, the remaining liquid (0.1%), which consists of exhaled droplets, has entrapped nonvolatile compounds at extremely low concentration. Each 1 mL of EBC contains <0.1 *μ*L of airway lining fluid (ALF) droplets [25]. Droplets are detached from ALF covering the surface of the respiratory tract due to the turbulent flow of the exhaled air through the airways [16, 26]. However, during exhalation, droplets are detached not only from the bronchi and trachea but also from larynx, pharynx, and upper gastrointestinal, nasal, and oral cavity and therefore it is impossible to identify the exact origin of the biomarkers [2]. Volume and number of exhaled droplets increase because of cough and increased secretion, while no correlation between ventilation and the amount of exhaled droplets has been described yet [24]. According to a recent hypothesis, nonvolatile compounds coming from the ALF undergo aerosolization during inhalation when bronchioles and alveoli burst to open (bronchiole fluid film burst (BFFB)) [27]. This fluid has previously been accumulated and trapped in the collapsed alveoli during exhalation [25, 28].

The concentration of nonvolatile compounds diluted in EBC is expressed by the equation of Effros et al. [24]:

(1) $$\begin{array}{c} {\left[\;X\;\right]}_{EBC}={\left[\;X\;\right]}_{ALF}\frac{V_{ALF}}{V_{EBC}}={\left[\;X\;\right]}_{ELF}\frac{n{\bar{V}}_{ALF}}{n{\bar{V}}_{ALF}+V_{vapor}}, \end{array}$$

where *V*_ALF_ = volume of exhaled droplets, *V*_EBC_ = volume of collected EBC, *V*_vapor_ = volume of condensed vapor in EBC, $n{\bar{V}}_{ALF}$ = number of respiratory droplets, and ${\bar{V}}_{ALF}$ = mean volume.

According to Effros et al. [24], the following parameters are responsible for the increase of substances in EBC:

- increased concentration of substances in the ALF,
- increased number and volume of droplets,
- reduced volume of water.

Based on the above, changes in levels of EBC biomarkers reflect changes in cellular composition and function [29–31].

#### 2.2.4. Dilution of EBC

EBC is a matrix containing large amount of water vapour [31] and thus most biomarkers are highly diluted, near the lower assay sensitivity limits. Given the fact that EBC dilution is higher than ALF dilution [32], it is necessary to establish an EBC dilution indicator able to reflect the actual levels of each biomarker in the airways. According to Effros et al. [24], an ideal dilution factor would have a known and stable plasma concentration and high diffusing capacity through the cellular membrane and would not be a product of the respiratory tract. Until now, no gold standard dilution factor has been defined and used extensively in studies. Nevertheless, multiple dilution indicators have been proposed, such as urea [30, 34–36], total protein [36], total cations (Na^+^, K^+^, Ca^2+^, Mg^2+^), and conductivity of lyophilized and vacuum-evaporated samples [30, 34].

#### 2.2.5. Sample Contamination

Since the main target is to detect compounds coming from the lower respiratory tract, exclusion of sample contamination by mediators and proteins originating from saliva, oral cavity, and upper airways is necessary [37]. EBC contamination can be avoided with the use of various methods, including saliva trap, swan-neck shaped tube, sodium bicarbonate 4,5% mouthwash prior to sampling, and periodic swallowing by the subject during EBC collection [1, 25]. To exclude salivary contamination after sampling completion, measurement of salivary amylase in EBC has been proposed [30, 38]. When the abovementioned precautionary measures are taken, amylase is barely detectable in EBC, found in 10.000-fold lower concentration compared to the saliva [30, 38].

### 2.3. Prior to Sampling

Exercise withdrawal is recommended 1 hour prior to the sample collection, since it may be responsible for changes in EBC compounds concentration [1]. Additionally, subjects are recommended not to smoke for 3 hours prior to EBC collection, as smoking has immediate effects on EBC-H_2_O_2_, 8-isoprostane, and NO-metabolites concentration [39, 41]. Caffeinated drinks must also be avoided when measuring EBC-adenosine levels [41]. Consumption of 1 L carbonated beverage or water prior to sampling causes significant reduction of EBC pH [42]. Finally, food consumption has not been shown to have any influence on EBC biomarkers concentration [41].

### 2.4. Sample Maintenance

Collected EBC must undergo instant analysis or it must be preserved at −70°C. Maintenance duration is determined by the stability time of chemical compounds concerned. Furthermore, repetitive freezing-defreezing cycles must be avoided, since this procedure results in loss of unstable chemical compounds [43].

### 2.5. Sample Processing

#### 2.5.1. pH Measurements

EBC pH is not stable, due to the significant pH reduction caused by both the atmospheric and the exhaled dissolved carbon dioxide (CO_2_). Therefore, avoidance of CO_2_ (gas standardization) is recommended in order to achieve accurate and comparable pH measurements [44]. CO_2_ deaeration is achieved either through bubbling free gas (usually argon) in order to displace CO_2_ or through bubbling a known quantity of CO_2_ into EBC samples, in order to standardize partial CO_2_ pressure to 40 mmHg. In both methods, pH measurement is recommended to be performed twice, before and after gas standardization, in order to confirm the pH change after CO_2_ deaeration [45, 46]. During argon deaeration, pH increases along with decreased pCO_2_, whereas during CO_2_ loading, pH is expected to decrease with a simultaneous increase in pCO_2_ [44].

#### 2.5.2. Prechromatographic Sample Processing

Chemical compounds extraction and concentration are required in order to analyze EBC sample with chromatographic methods. This procedure is achieved through various techniques, including (i) immunoaffinity extraction (IAE), in which special antibodies are used [47], (ii) solid phase extraction (SPE), which is based on chemical and natural properties of the chemical compounds [48], (iii) solvent extraction (SE), which is based on the relative water or organic solubility [49], and (iv) lyophiliosis, which requires exsiccation of the sample, achieved through cooling [50]. Lyophiliosis provides both volatile and semivolatile compounds concentration, but also removal of volatile compounds such as ammonia (NH_3_) [51]. Lyophilized EBC gives 20 times the concentrations of biomarkers, in comparison to the other techniques mentioned above [52].

#### 2.5.3. EBC-Analysis Methods

Various methods able to detect different compounds are available for EBC-analysis. Commercial ELISA kits are of low sensitivity and specificity, since they are not oriented to detect compounds at the extremely low concentrations identified in EBC. Furthermore, EBC itself is not an appropriate matrix for such commercial kits [53].

Therefore, other traditional techniques, including spectrophotometry [54], spectrofluorimetry [33], and enzyme based techniques [55], and immunoassay methods, such as ELISA, radioimmunoassay (RIA), immune sensors, and multiple immunoassay (MIA) [56] are frequently used. However, analysis based on the abovementioned techniques must be validated using analytical methods, such as mass spectrometry and high-performance liquid chromatography, able to provide quantitative analysis of EBC compounds [57].

Recently introduced methods, such as 2-dimensional protein gel electrophoresis (2D PEG) [57], followed by chromatographic proteomic microanalysis, are able to provide both qualitative and quantitative EBC-analysis. Contemporary chromatographic methods include gas chromatography (GC), high-performance liquid chromatography (HPLC), mass spectrometry (MS), and combinations of the abovementioned techniques, including LC-MS, GC-MS [58], HPLC-MS [59], Chromatography-Differential Mobility Spectrometry (GC-DMS) [60], and Electrospray Ionization-Differential Mobility Spectrometry (ESI-DMS) [61].

## 3. Clinical Applications of EBC-Analysis

### 3.1. Asthma

Numerous pioneering studies, enrolling relatively small number of subjects, detected significantly lower EBC pH in patients with stable asthma in comparison to healthy controls [62–65], and further pH reduction was identified during asthmatic exacerbation [62, 66–68]. Furthermore, EBC pH has been found significantly increased after treatment with inhaled corticosteroids [66, 69, 70]. However, it must be pointed out that, in big cohorts, no difference was demonstrated in EBC-pH between patients with stable asthma and healthy controls [45, 71–73], coming into conclusion that older studies demonstrated misleading findings.

Another marker of oxidative stress, hydrogen peroxide (H_2_O_2_), has been found in increased levels in EBC of asthmatic patients and significant correlation between H_2_O_2_ increase and forced expiratory volume in 1 sec. FEV_1_ reduction has been detected [74]. According to a meta-analysis by Teng et al. [75] based on 8 studies involving 500 patients with asthma and 228 healthy subjects, EBC-H_2_O_2_ levels were significantly higher in nonsmokers with asthma compared to healthy subjects. In particular, adults with asthma had higher EBC-H_2_O_2_ levels than healthy subjects. However, no significant difference was found between EBC-H_2_O_2_ concentration in children with asthma and control group. Moreover, EBC-H_2_O_2_ levels correlated with disease severity and control status, whereas an inverse correlation with FEV_1_ was found. Finally, treatment with corticosteroids resulted in a reduction of H_2_O_2_ levels. These findings indicate that H_2_O_2_ has a potential as a biomarker for guiding asthma treatment.

Leukotrienes (LTs) are well-studied inflammatory mediators, produced by leukocytes through arachidonic acid oxidation mediated by 5-lipoxygenase [76]. CysLTs (LTD_4_, LTE_4_, and LTC_4_) are produced from mast cells and eosinophils and they have been found increased in atopic patients [77] and patients with exercise-induced bronchoconstriction (EIB) [77, 78]. More specifically, preexercise levels of CysLTs have been found elevated in EBC of asthmatic patients with EIB (median concentration 42.2 pg/mL, associated with the decrease in FEV_1_ after exercise) compared to asthmatics without EIB (11.7 pg/mL) and healthy controls (5.8 pg/mL) [78]. Furthermore, another study demonstrated increased levels of CysLTs in asthmatic patients with EIB, 10 min after exercise [79]. This fact supports the concept that CysLTs release is involved in the development of EIB. LTs are released from the airway inflammatory cells after exposure to allergens, promoting bronchoconstriction, increase of vascular permeability, and mucus production [80]. Increased concentrations of LTs in EBC of asthmatic patients have been identified in numerous studies [15, 77, 81–88], and EBC-LTs levels increase is correlated with severity of the disease [89]. Moreover, treatment with oral corticosteroids during asthmatic exacerbation is followed by significant reduction of EBC-cysLTs (−60%) [90]. However, it has been shown that EBC-LTE_4_ levels are not significantly reduced after inhaled corticosteroid treatment in patients with stable asthma [83].

Additionally, increased levels of LTB_4_ have also been identified in patients with COPD [91], CF [92], and non-small cell lung cancer (NSCLC) [93] and thus, it is not an asthma-specific biomarker.

EBC-concentrations of other eicosanoids, such as 8-isoprostane and prostaglandin E_2_ (PGE_2_), have also been studied in asthmatic populations. 8-isoprostane levels have been found increased in adult and pediatric populations with stable asthma, but they were not significantly reduced after inhaled or oral corticosteroid treatment [83, 94, 95]. EBC-PGE_2_ concentration was not increased significantly among both children and adult nonsmokers with stable asthma [15, 83, 95]. However, levels of EBC-PGE_2_ have been found significantly increased in adult smokers with stable asthma [96]. Therefore, increased EBC-PGE_2_ possibly reflects smoking status in asthmatic patients.

There is also detailed literature regarding EBC-interleukins (ILs) concentration in patients with asthma, detecting increased levels of IL-4 [97–102], IL-5 [103], IL-6 [98–101, 104], IL-8 [105], IL-10 [45], and IL17 [105]. In a study of Shahid et al. [97], increased levels of EBC-IL-4 with simultaneous reduction of interferon-*γ* were identified among pediatric asthmatic population, changes indicative of Th2-mediated inflammation.

Another interesting biomarker involved in bronchoconstriction mechanism is RANTES. It is a chemotactic agent for eosinophils, T-lymphocytes, and mast cells, which induce histamine and Cys-LTs secretion from mast cells and eosinophilic cationic protein from eosinophils, causing bronchospasm in patients with asthma [106]. In a study of Matsunaga et al. [105], simultaneous increase of IL-4, IL-6, IL-8, IL-10, TNF-*α*, TGF-*β*, MIP1*α* (Macrophage Inflammatory Protein 1*α*, CCL3), MIP1*β* (Macrophage Inflammatory 1*β*, CCL4), and RANTES was identified among adult steroid-naïve asthmatic patients. Furthermore, EBC-RANTES increase was significantly correlated with both FEV_1_ reduction and airways resistance increase, while increased levels of EBC-TNF-*α* and TGF-*β* were significantly correlated with nonspecific bronchial hyperresponsiveness (BHR, methacholine challenge).

In other studies by Zietkowski et al. [107, 108] significantly increased levels of EBC-RANTES and endothelin-1 (ET-1) were identified in adult patients with stable asthma compared to the healthy controls. Moreover, both RANTES and ET-1 levels were significantly higher in subjects with unstable asthma, compared to stabilized asthmatic subjects. Therefore, it seems that EBC-RANTES levels are correlated with severity of the disease.

Extensive proteomic analysis of EBC has also revealed other biomarkers related with pathophysiological mechanisms involved in asthma. In a study of Bloemen et al. [109], significantly higher levels of EBC-Cytokeratine 1 were detected among asthmatic children compared to healthy controls, whereas no significant differences were identified in concentrations of EBC-cytokeratines 2, 5, 6, 8, 9, 10, 14, and 16, albumin, actin, haemoglobin, lysozyme, calgranulin B, and desmin. In a study of Bartoli et al. [110], EBC-Malondialdehyde (MDA) levels were found significantly higher among asthmatic patients in comparison to healthy controls (26 nM versus 15.6 nM, resp.), and inhaled corticosteroid treatment was related to lower EBC-MDA levels (21 nM) in comparison to untreated patients (32 nM). However, EBC-MDA levels were not significantly correlated with severity of the disease in this study.

Asymmetric Dimethylarginine (ADMA) is an L-arginine analog, which inhibits nitric oxide (NO) synthesis, contributing to asthma pathogenesis (airways inflammation and remodeling, oxidative stress, and BHR). In a study of Carraro et al. [111], significantly increased levels of EBC-ADMA/Tyr ratio (median value = 0.12, interquartile range 0.05–0.32) have been identified among pediatric population, compared to healthy controls (median value = 0.07, interquartile range 0.05–0.12).

Eotaxin-1 (CCL11) is a chemokine for the eosinophils, inducing both their accumulation to the airway wall and their activation and degranulation. In a study by Zietkowski et al. [112], significantly increased EBC-CCL11 levels were identified among patients with stable asthma in comparison to healthy controls, and patients with unstable asthma demonstrated significantly higher CCL11 levels compared to stabilized asthmatic patients. Furthermore, significant reduction of CCL11 levels was identified after omalizumab treatment among patients with severe asthma, reflecting remission of eosinophilic inflammation [113].

In another study by Zietkowski et al. [114], significantly increased levels of EBC-high-sensitivity c-reactive protein (hs-CRP), a well-studied inflammatory mediator, were identified among patients with stable asthma compared to healthy controls, and significantly higher levels of EBC-hs-CRP were detected in patients with unstable asthma in comparison with stabilized asthmatics. Moreover, not only serum hs-CRP but also Fractional Exhaled Nitric Oxide (FeNO) levels were significantly correlated with EBC-hs-CRP levels in all subjects.

EBC genomic analysis is also becoming an intriguing area of research, especially when combined with proteomic analysis. In a prospective study of Klaassen et al. [115], intercellular adhesion molecule-1 (ICAM-1) genes expression in terms of mRNA and proteins was related to asthma manifestation among preschool children. Significantly increased EBC-solute-ICAM-1, as well as EBC-1L-4, IL-10, and IL-13, was found among children with asthma.

EBC has been also studied in asthmatic patients with GERD (gastroesophageal reflux disease) in order to assess the airway acidification. When the acid refluxes up to the pharynx, it enters the airways causing epithelial damage due to acid injury and therefore worsening of asthma symptoms. In a study investigating the determinants of EBC-pH in a large population with asthma, GERD symptoms were associated with low EBC-pH [116]. However, Niimi et al. [63], enrolling patients with chronic cough suffering from various diseases (asthma, rhinitis, GER, bronchiectasis) demonstrated that low EBC-pH was associated with chronic cough, regardless of the underlying medical condition. Therefore, low EBC-pH is not a specific finding for GER.

Banovic et al. [117] demonstrated that children with uncontrolled asthma had significantly lower mean EBC-pH than children with GERD without asthma (6.791 ± 0.374 versus 7.002 ± 0.361, *p* = 0.006). Moreover, both asthmatic and GERD children had lower total magnesium (10 *μ*mol/L and 20 *μ*mol/L, resp.) with the later demonstrating a negative correlation (*r* = −0.404, *p* = 0.003) between magnesium levels and pH values. Additionally, in a study by Fitzpatrick et al. [118] EBC-pH failed to distinguish patients with asthma depending on the presence of asymptomatic GERD. Furthermore, EBC-pH did not alter significantly after treatment with lansoprazole, failing to act as a marker for the response to PPIs treatment. However, in a former study by Shimizu et al. [119], patients with moderate asthma and GERD responded to a 2-month PPI therapy (lansoprazole 30 mg/day) by increasing pH (from 7.2 ± 0.1 to 7.3 ± 0.1) and significantly reducing 8-isoprostane levels (from 32.7 ± 3.4 to 19.2 ± 3.4) along with the improvement of GERD symptoms.

Pepsin was also used as a marker of GER-related aspiration in patients with GERD and GERD-induced respiratory problems. Pepsin is normally present in gastric secretions but not in bronchial tree, so an increase of EBC-pepsin would be expected in GERD patients. However, in a study by Soyer et al. [120], pepsin levels in EBC of GERD patients were below detection limits, failing to verify the abovementioned hypothesis. Nevertheless, in this study, EBC-NO_*x*_ (nitrites and nitrates) levels were lower in GERD patients compared with non-GERD patients (13.7 *μ*mol/L versus 21 *μ*mol/L). A negative correlation between reflux index and NO_*x*_ levels in EBC was also demonstrated (*r* = −0.331, *p* = 0.023). This finding indicates that longer periods of reflux may cause more epithelial damage and subsequently a drop in NO_*x*_ levels. Based on the above, there is no specific EBC biomarker indicative of airway acidification due to GERD in asthmatic patients.

Summarizing EBC alterations in asthma, it can be observed that the main problem of existing studies is the poor reproducibility of results. This could be attributable to the lack of specific analysis of the different inflammatory pathways in the vast majority of studies. Asthmatics with neutrophilic pattern of inflammation would possibly have mediators in their EBC different from asthmatics with an eosinophilic pattern and probably would have a different response to therapy. Therefore, existing knowledge is insufficient regarding the detection of specific markers for asthma.

An additional methodological issue is that, in the great part of studies, biomarkers have been studied only in stable asthma (Table 1). More studies concerning biomarkers in unstable asthma are needed, aiming to predict exacerbation and treatment response, especially to corticosteroids, which is of paramount importance in daily clinical practice (Table 1 summarizes the changes of exhaled breath condensate biomarkers in asthma).

### 3.2. Chronic Obstructive Pulmonary Disease (COPD)

#### 3.2.1. Immediate Effects of Tobacco Smoking on EBC

Immediate effects of smoking a single cigarette (CS) have been extensively studied. Some early studies detected nonsignificant changes of EBC-pH among healthy smokers [121, 122], whereas, in a recent study, immediate increase of EBC-pH was identified, lasting for 2 hours after CS [41]. Interestingly, the opposite change (significant reduction) in EBC-pH was identified immediately after CS among patients with allergic rhinitis [123] and asthma [121, 124], which is indicative of a different acute response of the respiratory system after smoking, probably reflecting the additive effects of smoking and atopic airway inflammation expressed as increased oxidative stress.

Significant increase of EBC-nitrates concentration (from 20,2 *μ*M to 29,8 *μ*M) [39], EBC-8-isoprostane levels (+50%) [40], and EBC-electric conductivity (from 40 *μ*S/cm to 90 *μ*S/cm) [122] immediately after CS has also been identified. However, a recent study demonstrates nonsignificant 8-isoprostane change [121].

In regard to EBC-nitrites, S-nitrosothiols, nitrotyrosine [39], ammonium, and IL-8 [122] levels, no significant change has been demonstrated among healthy smokers immediately after CS.

A recent study investigating the immediate effects of 30-minute waterpipe smoking (WPS) on EBC [125] detected significant reduction of EBC-IL-4, IL-5, IL-10, IL-17, and INF-*γ*, due to either decreased production or decreased delivery, as an effect of high levels of carbon monoxide (CO), which is a potent anti-inflammatory agent.

#### 3.2.2. Long-Term Effects of Tobacco Smoking on EBC

It has been shown that there is no significant difference in EBC-pH among mild healthy smokers (<10 pack-years) and healthy never-smokers (median values: 8.17 versus 8.16, resp.). Contrarily, EBC-pH of asymptomatic smokers with cigarette consumption higher than 10 pack-years has been found significantly reduced, presenting values close to the ones of COPD patients (median values: 7.4 and 7.36, resp.) [122, 126]. Furthermore, pH reduction is significantly correlated with smoking history (0.05-degree EBC-pH reduction for every year of regular cigarette smoking was identified) [126].

Inflammatory agents, including EBC-IL-6, LTB_4_ [116], IL-8 [127, 128], and TNF-*α* [129], have been found significantly increased in chronic, asymptomatic smokers. Moreover, EBC-IL-6 and TNF-*α* levels are significantly correlated with Forced Vital Capacity (FVC) and FEV_1_ values [127, 129], while levels of exhaled CO (eCO) and smoking history are significantly correlated only with IL-6 levels [129]. Additionally, it has been shown that smokers with allergic rhinitis demonstrate increased baseline EBC-LTB_4_ levels [130], suggesting that rhinitic smokers are more susceptible to the deleterious effects of smoking.

#### 3.2.3. Secondhand Smoking (SHS) Effects

Little is known regarding SHS effects on EBC. In a recent study [131], significant reduction of EBC-pH was identified among healthy nonsmokers immediately after 1 hour of exposure to SHS, lasting for 180 minutes. Moreover, significantly increased levels of EBC-H_2_O_2_ were identified at 120 minutes after exposure and remained increased for the next 120 minutes.

There are still a limited number of studies investigating the effects of cigarette smoking on EBC. However, it has been proved that even a single cigarette smoking causes significant alterations involving inflammatory pathways. Furthermore, there is a different inflammatory response (both short- and long-term) of the respiratory system to cigarette smoking among smokers with and without allergic rhinitis. This finding indicates that there is a high possibility that the same could apply to smokers with and without asthma. Carefully designed studies are needed in order to investigate this possibility and the susceptibility of these patients to develop COPD in the future (Table 2 summarizes the short- and long-term effects of tobacco smoking on EBC).

#### 3.2.4. Stable COPD

EBC-pH of COPD patients has been found acidic in numerous studies [57, 132, 134], reflecting the endogenous airway acidification. Furthermore, significant correlation between pH reduction and FEV_1_ decrease has been identified [57], but this finding has not been confirmed by other investigators [132]. Influence of smoking was determined in a large cohort by MacNee et al. [133], who demonstrated that EBC pH was lower in COPD than in healthy control nonsmokers but did not differentiate COPD from smokers without COPD. According to these findings, EBC pH does not appear to be a useful biomarker in smokers with COPD.

In another study, Papaioannou et al. [134] detected significantly reduced EBC-pH in COPD patients, both smokers and ex-smokers, in comparison to healthy controls (both smokers and ex-smokers). Interestingly though, ex-smokers of COPD patients demonstrated significantly lower EBC-pH values than their still-smoking counterparts. Additionally, ex-smokers with GOLD stages III and IV COPD demonstrated significantly reduced EBC-pH compared to ex-smokers with GOLD stages I and II COPD, whereas no significant difference was detected among smokers of COPD patients with increasing GOLD stage. Furthermore, EBC-pH levels were correlated with static hyperinflation (IC/TLC (Inspiratory Capacity/Total Lung Capacity)), air trapping (RV/TLC (Residual Volume/Total Lung Capacity)) and diffusing capacity for carbon monoxide in ex-smokers, whereas no corresponding significant correlations were identified among current smokers.

Based on the above, current data regarding EBC-pH in COPD is conflicting. The biomarker may be used as a COPD-severity index only in ex-smokers of COPD patients, since it seems that the inflammatory effects of current smoking, bursting the airway oxidative stress, cover up the ongoing inflammatory process of the disease.

In regard to airway oxidative stress, EBC-H_2_O_2_ has been found significantly increased among COPD patients in numerous studies [135–137], but there was no significant correlation between EBC-H_2_O_2_ increase and FEV_1_ reduction [135].

Furthermore, in a study of Corradi et al. [138], EBC-concentrations of various aldehydes (MDA, hexanal, heptanal), well-characterized biomarkers of the attack of Reactive Oxygen Species (ROS) on unsaturated lipids in cell membranes, were found significantly increased in COPD patients compared to healthy nonsmokers. However, only EBC-MDA concentration (significantly increased in COPD) served as a distinguishing factor between smoking controls and COPD patients, suggesting that MDA could be a potentially useful biomarker in COPD patients follow-up. Another study of Corradi et al. [139] demonstrated increased levels of aldehydes (mainly MDA) in EBC of COPD patients compared to control groups, whereas in a more recent research by Antus et al. [140] difference in MDA levels between COPD patients and healthy controls was found in sputum but not in EBC.

In regard to classic inflammatory markers, EBC-8-isoprostane levels have been found equally increased in both smokers and ex-smokers of COPD patients, compared to healthy controls [136, 141, 142] (1,8-fold in COPD versus healthy smokers and 2,2-fold in COPD versus healthy nonsmokers [136]). However, no significant correlation among EBC-8-isoprostane increase and FEV_1_ reduction was identified. Significant increase of EBC-LTB_4_ has been detected among smokers with COPD in comparison to healthy smokers [16, 141] and significant correlation among EBC-LTB_4_ increase and CO diffusing capacity reduction has been identified, whereas EBC-LTB_4_ increase was not correlated with FEV_1_ reduction [141]. Additionally, no significant difference in levels of EBC-LTB_4_ among COPD smokers and ex-smokers has been detected [142]. Thus, it seems that increased levels of 8-isoprostane and LTB_4_ may be indicative of increased airway inflammatory stress regardless of smoking status. However, Borrill et al. [143] observed considerable within- and between-day variability of these biomarkers, posing limitations to the value of the abovementioned findings.

Increased levels of EBC-PGE_2_ and PGF_2_ have also been identified in COPD patients compared with asthmatic patients. However, significantly increased levels of EBC-LTE_4_ and TxB_2_ have been detected in asthmatic patients, whereas the abovementioned biomarkers were undetectable in COPD patients [16].

A special characteristic of airway inflammation in COPD is its mediation by neutrophils, activated through interaction with cytokines, especially interleukins (ILs). Indeed, significantly increased levels of IL1*β* and IL-12 have been found in patients with stable COPD [52]. Furthermore, in a recent study of Fumagalli et al. [144], where proteomic EBC-analysis was applied, different EBC proteomic profiles were identified among COPD patients and healthy controls, regardless of smoking status. More specifically, signaling and regulatory proteins, including cytokeratins I and II (CK-1, CK-5, CK-9, CK-14, and CK-26), were dominant (34%) in EBC of healthy controls (smokers and nonsmokers). In COPD patients, including patients with *α*_1_-antitrypsin deficiency (A_1_AD), cytokines (IL-1*α*, IL-1*β*, IL-2, IL-12*α*, IL-12*β*, and IL-15) were identified as the dominant protein type (62%), but INF-*α*, INF-*γ*, TNF-*α*, and C3 complement fraction were detected. In healthy controls, levels of the abovementioned inflammatory mediators were negligible and INF-*α* and IL-12 were not detected. Interestingly though, haemoglobin *β*-chain was detected in healthy smokers, probably reflecting haemolysis due to smoking induced oxidative stress. Additionally, considerable amount of enzymes (8%) which were identified in healthy controls, was absent in COPD patients. Furthermore, COPD versus A_1_AD comparative EBC proteomic analysis, demonstrated significant differences. In A_1_AD-patients, *α*_1_-antitrypsin was not detected, but IL-1*α* and lysozyme-C, which were undetectable in the other COPD patients, were identified. Other types of proteins, including surfactant proteins (SP-A1, SP-A2) and S100 calgranulin A and calgranulin B (calcium-binding proteins), were equally identified in both healthy controls and COPD patients, but not in A_1_AD-patients. The abovementioned differences in proteomic profile may prove to be a useful tool in regard to identification of pathogenetic mechanisms involved in COPD.

#### 3.2.5. Acute COPD Exacerbation (AECOPD)

EBC-pH has been found significantly reduced during AECOPD (5.57 ± 0.07) and significant increase following treatment has also been identified (6.04 ± 0.08) [145]. Additionally, increased levels of EBC-CysLts [3], LTB_4_ [146, 147], 8-isoprostane, and H_2_O_2_ have been detected, followed by significant decrease after antibiotic treatment with cephalosporins and macrolides [146].

In regard to classic inflammatory markers, significantly increased levels of EBC-IL-6 [52, 148], IL-1*β*, IL-8, IL-10, IL-12, TNF-*α* [52], and EBC-nitrites [149] have been identified in patients with AECOPD, in comparison to patients with stable COPD.

EBC proteomic analysis in patients with infectious AECOPD revealed significant increase of *α*_1_-antitrypsin-antiprotease (AAT, 2-fold in comparison to stable COPD) [150], metalloproteinase-9 (MMP-9), and metalloproteinase-1-inhibitor (TIMP-1) [151], reflecting the acute phase response. In a study of Corhay et al. [152] which enrolled ex-smokers with COPD, EBC-neutrophil chemotactic activity (NCA, assessed by the use of Boyden microchambers) was found increased in outpatients with AECOPD, followed by significant reduction 6 weeks after AECOPD recovery. However, no significant increase of EBC-NCA was detected in hospitalized patients with AECOPD and no significant difference on EBC-NCA was identified between patients with stable COPD and outpatients with AECOPD. In regard to neutrophil chemoattractants, EBC-LTB_4_ was found significantly increased in patients with both stable COPD and AECOPD, but EBC-GRO-*α* (growth related oncogene-*α*) was found significantly increased only in AECOPD patients. Moreover, EBC-LTB_4_ and EBC-GRO-*α* reduced significantly after recovery from AECOPD. Based on the above, GRO-*α*, a strong neutrophil chemoattractant, may be pathogenetically involved in AECOPD and further investigation is necessary in order to evaluate its role in the follow-up of such patients.

Other agents have been also proposed as prognostic factors of AECOPD, including increased levels of solute-HLA-1 and solute-CD95 [153]. Contrarily, in other studies demonstrating increased levels of EBC-secretory leukocyte protease inhibitor-1 (SLP-1) and myeloperoxidase, the authors concluded that such changes probably reflect the pulmonary immune response to antibiotic treatment, rather than a pathogenetic mechanism involved in manifestation of AECOPD [154].

EBC genomic analysis has also given interesting results. Nucleic acids of both viral and bacterial agents, including influenza virus and respiratory syncytial virus, but also Legionella pneumophila, have been detected in EBC of AECOPD patients, while EBC analysis has been proved more sensitive in detection of Legionella pneumophila compared to induced sputum [155].

Summarizing current knowledge in COPD, there are numerous studies investigating a variety of EBC biomarkers in both stable and exacerbated COPD, without promising results regarding a reliable predictor of exacerbation. Furthermore, no study demonstrates specific inflammatory pathways that can differentiate between and ideally predict COPD clinical phenotypes (i.e, chronic bronchitis, emphysema, overlap syndrome of asthma, and COPD). Further research is needed in order to identify the specific fingerprint of each phenotype which would lead to different treatment approaches (Table 3 summarizes the changes of EBC biomarkers in COPD).

### 3.3. Lung Cancer

There is a lot of potential regarding the role of EBC-analysis in early diagnosis, prognosis, as well as follow-up of patients with lung cancer, since a plethora of tumor-specific EBC biomarkers have been identified to date.

In regard to oxidative stress, Lases et al. [156] detected significantly higher increase of EBC-H_2_O_2_ and urine-MDA in patients with lung carcinoma, who had undergone lobectomyin comparison to pneumonectomy, but also a strong correlation was found between EBC-H_2_O_2_ and urine-MDA.

In regard to inflammatory markers, Carpagnano et al. [157] first detected increased levels of EBC-IL-6 in patients with non-small cell lung cancer (NSCLC) compared to healthy controls. In another study of Carpagnano et al. [158] increased levels of EBC-IL-2, TNF-*α*, and leptin were found in patients with stages I and II NSCLC compared with healthy ex-smokers. In a study of Brussino et al. [159], increased EBC-IL-6, IL-17, and TNF-*α* were associated with increased levels of EBC-Vascular Endothelial Growth Factor (VEGF) and all the abovementioned biomarkers were significantly correlated with the tumor diameter, estimated through CT. Additionally, Carpagnano et al. [160] have also detected increased levels of EBC-endothelin-1 (ET-1) in NSCLC patients, and Dalaveris et al. [161] demonstrated increased TNF-*α* and VEGF in patients with T3 and T4 NSCLC.

However, many of the abovementioned inflammatory markers have also been found in increased levels among patients with COPD and asthma, as previously mentioned.

Kullmann et al. [162] demonstrated that the cytokine profile in EBC of patients with lung cancer was different from that of controls regardless of smoking habits, lung function, and airway inflammation. More specifically, EBC-analysis of lung cancer patients showed 3-fold increase of CCL-28 (lymphocyte chemoattractant), 2-fold increase of MIP-3 (granulocyte activator) and GRo-a (monocyte adhesion molecule), 3-fold decrease of FGF-6 (fibroblast growth factor), 2.5-fold decrease of eotaxin-2, eotaxin-3 (eosinophil chemoattractants), FGF-7 (fibroblast growth factor), and IL-10 in comparison to control group. However, in a more recent study of Barta et al. [163], no difference was found between patients with squamous cell lung carcinoma and healthy smokers based on EBC cytokine signals.

Furthermore, significantly increased levels of proinflammatory mediators, including EBC-IL-1*β*, IL-6, IL-8, TNF-*α*, and sICAM-1, have been measured on postoperative days (days 1, 3, and 7) in patients who have undergone lobectomy or pneumonectomy [164]. sICAM-1 was significantly higher on day 1 after resection (sICAM-1 is associated with malignant tumours), TNF-*α* remained increased from day 1 to day 7, IL-8 peaked on day 3, IL-6 peaked on day 1, and IL-1*β* levels increased after day 1. There was no significant association, however, between levels of EBC-IL-1*β*, IL-6, IL-8, TNF-*α*, and sICAM-1 and the extent of lung cancer resection (lobectomy versus pneumonectomy) [164].

In a study by Gessner et al. [165], significantly increased levels of EBC-VEGF and bFGF (basic fibroblast growth factor) were identified in patients recently diagnosed with NSCLC, compared to healthy controls, patients with stable COPD, and patients with AECOPD. Furthermore, significantly lower EBC-levels of the abovementioned growth factors were identified among patients who underwent 2-cycle chemotherapy and who responded with 25% reduction of the tumor size, compared to recently diagnosed untreated patients.

Further EBC proteomic analysis has given numerous promising markers, still requiring further evaluation. Carpagnano et al. [166] suggested that EBC-COX-2 (cyclooxygenase-2) and survivin, an apoptosis inhibitor, could be used as markers for early lung cancer diagnosis among high risk smokers, since significantly increased levels of EBC-COX-2 and survivin were identified in healthy smokers compared to nonsmokers, but also in NSCLC patients compared to healthy smokers. Moreover, increased levels of the abovementioned biomarkers were associated with increasing stage of NSCLC.

In another study by Carpagnano et al. [167] high concentration of EBC-matrix metalloproteinase-9 (MMP-9) has been found in NSCLC patients and quantitative correlation was identified between EBC-MMP-9 levels and smoking history, but also with the stage of the disease.

Cheng et al. [168] first identified the Growth Hormone Regulated TBC Protein 1 (GRTP-1) in EBC of a single patient with NSCLC, a finding that warrants further evaluation, as suggested by the authors themselves. Moreover, out of a total number of 29 proteins detected, 18 were common among healthy nonsmokers, healthy smokers, and NSCLC patients, 1 was common among healthy smokers and NSCLC patients and 4 were identified only in ex-smokers.

Zou et al. [169] suggested that EBC-CEA (carcinoembryonic antigen), SCC (squamous cell carcinoma antigen), and NSE (neuron-specific enolase) were more sensitive indexes for diagnosis of early stage NSCLC and more strongly correlated to the histological type compared with the serum-CEA, SCC, and NSE. The authors concluded that EBC-CEA was the parameter with the best diagnostic accuracy in regard to NSCLC, especially for adenocarcinoma (Se = 83.8%, Sp = 67.9%). EBC-SCC and EBC-NSE also demonstrated acceptable distinction capacity (Se = 38.1%, Sp = 92.9%, Se = 55.2%, and Sp = 66.1%, resp.).

Increasing number of studies demonstrated impressive results after applying genomic analysis of EBC. Gessner et al. [170] detected somatic mutations of the* p53* gene in patients with lung cancer, which were undetectable in healthy controls. Carpagnano et al. [171] described microsatellite DNA instability, located in* 3p* chromosome (locus* D3S2338*,* D3S1266*,* D3S1304*, and* D3S1289*), but also loss of heterozygosity, both in healthy smokers and NSCLC patients. In another study conducted by the same team [172], comparative analysis of mutations in DNA isolated from EBC, peripheral blood, and tumor tissue detected superiority of EBC as a matrix for identification of DNA mutations in patients with NSCLC. In this study [172], the same microsatellite EBC-DNA changes were identified both in heavy smokers and patients with NSCLC.

An additional conclusion of the same authors was that the amount of detected 3p mutations was of negative prognostic value, since it was associated with poor survival of patients with lung cancer. More specifically, loss of heterozygosity in* D3S1289* demonstrated negative prognostic value for lung adenocarcinoma, but it was also related with poor prognosis of patients with NSCLC, regardless of the stage of the disease. Moreover, microsatellite instability and loss of heterozygosity in* D3S2338* were associated with poor survival of patients with squamous cell lung cancer [173].

Microsatellite EBC-DNA mutations have also been detected in chromosome* 19q*, resulting in* ERCC-1* and* ERCC-2* genes, which were present in 25% of NSCLC patients and 16% of smoking subjects, but they were undetectable in nonsmokers [174].

Mozzoni et al. [175] detected increased levels of* miRNA-21* and particularly reduced levels of* miRNA-486* in patients with NSCLC and proposed their use as first-line screening test for diagnosis of NSCLC.

Another impressive finding regarding lung cancer pathogenesis has been identified in a study of Kordiak et al. [176]. Specifically, levels of mutated* KRAS* oncogene (mutation located in codon 12) were significantly reduced in EBC of patients with NSCLC after tumor resection, by 1.3 times at 7th postoperative day and by 3,7 times at 30th postoperative day. In 10 patients the specific* KRAS* mutation was undetectable at 30th postoperative day.

DNA methylation is a common biochemical process, essential for normal development through various genomic functions, including X-chromosome inactivation, but also suppression of repetitive elements. However, aberrant DNA methylation patterns (hyper and hypomethylation) have been associated with numerous human malignancies. In a study of Han et al. [177], ex-smokers had higher density of methylated* RASSF-1A* gene compared to current smokers and nonsmokers and methylation of the* DAPK* and* PAX5β* genes was significantly correlated with NSCLC. In a latter study, Xiao et al. [6] detected methylation of* p16* gene in EBC of patients with NSCLC.

HPV (human papillomavirus) infection has been proposed as the main infectious agent involved in the pathogenesis of lung cancer, but it has been demonstrated that fungal infections could also be involved, through production of carcinogenic mycotoxins. In a study by Carpagnano et al. [178], HPV genome was identified in EBC of 16.4% of a total population with NSCLC, while it was undetectable in the total healthy control population. Furthermore, the same authors, using the technique of EBC cultivation for first time were able to identify that 27.9% of a total population with NSCLC was colonized with* Aspergillus niger*,* A. Ochraceus* or* Penicillium* spp., whereas fungal colonization was absent in EBC of the healthy control population [179].

In regard to lung cancer, there is rapidly increasing number of studies on EBC biomarkers applying contemporary techniques, especially proteomic and genomic analysis. The main goal of these studies was to demonstrate biomarkers for early diagnosis of LC. The most promising results came from genomic analysis in studies enrolling heavy smokers and NSCLC patients in which common DNA alterations were identified. This finding is promising and indicates that such alterations may be of prognostic value. However, methodological issues should be addressed: current knowledge is based on studies enrolling patients with NSCLC and there is a relative lack of studies enrolling patients with small cell lung carcinoma, which is also a prevalent disease. Additionally, large scale screening and prospective studies are needed, enrolling asymptomatic patients with all histological types of LC, in order to evaluate if EBC markers could identify a preclinical stage of the disease (Table 4 summarizes the exhaled breath condensate biomarkers in lung cancer).

### 3.4. EBC in Mechanically Ventilated Patients

EBC has been widely studied in mechanically ventilated (MV) patients as a noninvasive alternative to evaluate airway inflammation, predict the development of VAP (ventilator associated pneumonia), and assess the ventilator associated damage and lung distention. EBC biomarkers have been studied not only in MV patients with ALI/ARDS and VAP but also in MV patients without respiratory failure and ALI/ARDS (i.e, brain injured).

Acidic pH has been found in MV patients both with [180, 181] and without lung injury [182, 183] compared to healthy controls. Interestingly, in MV patients without ALI, EBC pH has been proved to inversely correlate with ventilation time (*r* = −0.636, *p* = 0.048) supporting the hypothesis that positive-pressure ventilation induces pulmonary inflammation and physical disruption of tissues and cells, resulting in airway acidification [184]. Additionally, EBC pH shows further reduction when MV patients' clinical condition deteriorates [181] or lung injury progresses [180] and returns to higher levels after recovery [181]. EBC pH values also elevate after salbutamol administration in MV patients with ALI (from 7.66 to 7.83) [185]. However, in a study by Nannini et al. [186], EBC pH failed to distinguish MV patients according to whether they eventually developed VAP, died, or met criteria for weaning.

A compound formed by in vivo peroxidation of arachidonic acid membrane, 8-iso-PGF_2a_, was found significantly higher in MV patients with ALI/ARDS (87 ± 28 pg/mL) compared to healthy subjects intubated during minor surgical procedures (7 ± 4 pg/mL) [187]. Additionally, reduction of 8-iso-PGF_2a_ was described after salbutamol inhalation along with a tendency to decrease nitrosative species [185]. These findings indicate that salbutamol might prevent the lipid peroxidation that characterizes ALI and ARDS. In a later study by Roca et al. [182] 8-isoprostane levels were not significantly different between MV patients without ALI and healthy nonsmokers.

The hypothesis that neutrophil activation and oxidant production are involved in the pathogenesis of ARDS is supported by several studies. MV patients who eventually developed ARDS demonstrated higher levels of H_2_O_2_ (1.68 ± 0.35 mumol/L) compared to others who did not (0.34 ± 0.08 mumol/L) [188]. Other inflammatory biomarkers, such as IL-1*β*, IL-10, IL-12p70, and TNF-*α*, are also elevated in EBC of MV patients both with and without ALI/ARDS, revealing the airway inflammation in such patients [183, 189], with the former having also increased IL-6 and IL-8 [189]. LTB4 and H_2_O_2_ have been found significantly elevated in MV patients who underwent lobectomy, but this change was not significant in MV patients after coronary artery by-pass graft surgery, probably due to the higher risk of ALI development after lobectomy [190]. In a study by Gessner et al. [191] EBC-NO_2_^−^ increased linearly with *V*_*T*_ and correlated well with expiratory minute volume, possibly due to mechanical stress of the remaining open lung units in injured lungs.

NO_2_^−^/NO_3_^−^ were found elevated in MV patients without ALI (mean 66.22 *μ*M) compared to healthy subjects (mean 15.06 *μ*M) and their levels had negative correlation with dynamic compliance (*r* = −0.952, *p* < 0.001) and positive correlation with respiratory rate (*r* = 0.683, *p* = 0.029) [182]. This finding could be related to the mechanical stress induced by positive-pressure mechanical ventilation.

Cytokeratins 2, 9, and 10 were also detected in EBC of MV patients with respiratory failure. PEEP, PIP, and LISS (lung injury score) were positively correlated with the levels of EBC-cytokeratins (2, 9, and 10), revealing a relationship between these biomarkers and the ventilator associated damage to the lung parenchyma [192]. sTREM-1 (soluble triggering receptor expressed on myeloid cell-1) was detected both in BAL and EBC of MV patients with VAP, indicating that the patients respond to bacterial stimuli with the triggering of the secretion of inflammatory cytokines by sTREM-1 [193].

### 3.5. Other Lung and Systemic Diseases

#### 3.5.1. Idiopathic Pulmonary Fibrosis (IPF)

Chow et al. [194] demonstrated significantly elevated pH and increased levels of 8-isoprostane, H_2_O_2_, 3-nitrotyrosine, and nitric oxides (NO_*x*_) in patients with IPF, compared to healthy controls.

Additionally, in a study by Montesi et al. [195], 9 different types of lysophosphatidic acid (LPA), which is an important mediator of fibroblast requirement, were detected in EBC of IPF patients. Moreover, docosatetraenoyl-LPA was found significantly increased in comparison to healthy controls (9.18 pM versus 0.34 pM, resp.).

#### 3.5.2. Cystic Fibrosis (CF)

In a study by Tate et al. [196], significantly reduced EBC-pH was detected in patients with CF compared to healthy controls, but also further pH reduction was identified during CF exacerbation. However, in a recent research by Antus et al. [197], pH does not appear to be a useful biomarker in CF, since it does not discriminate between CF patients and healthy controls.

A significant increase of EBC-nitrotyrosine was identified in patients with stable CF but there was no significant correlation between FeNO and EBC-nitrotyrosine levels [198]. However, this finding was not confirmed by a later study by Celio et al. [199], who showed no difference in EBC 3-nitrotyrosine levels between CF patients and healthy controls.

In a study by Horak et al. [200], increased levels of EBC-nitrite were not associated with either lung function or chest X-ray findings. Those results are in contrast with a latter study of Robroeks et al. [201] who proposed increased EBC-nitrites and 8-isoprostane as markers of CF exacerbation. They also found a reduction of EBC-nitric oxide and pH which was correlated with severity of the disease.

In regard to classic inflammatory markers, Cunningham et al. [202] demonstrated no significant difference in EBC-IL-8 levels among CF patients and healthy controls. However, in a latter study by Carpagnano et al. [203], significantly increased levels of EBC-IL-6, IL-8, and LTE_4_ were identified during CF exacerbation, especially when it was induced by* Pseudomonas aeruginosa* infection. Moreover, significant reduction of the abovementioned inflammatory markers was detected after treatment with antibiotics [203, 204]. In a study by Robroeks et al. [205], no significant difference was detected in EBC-IL-1, IL-4, IL-10, and INF-*γ* concentration among asthmatic and CF paediatric populations, whereas EBC-IL-5 and TNF-*α* were detectable only in the CF-population.

#### 3.5.3. Pulmonary Arterial Hypertension- (PAH-) Influence of COPD

In a study by Warwick et al. [206], significantly higher levels of EBC-natriuretic peptide, pro-BNP, and ET-1 were identified in patients with idiopathic PAH (IPAH) compared to patients with COPD-induced PAH, whereas in the latter, significantly higher levels of 6-keto-PGF_1*α*_ were detected. Additionally, He et al. [207] demonstrated a significant raise of EBC-8-isoprostane and IL-6 in COPD patients with PAH in comparison to patients with IPAH.

In a study by Carratu et al. [208], significantly higher EBC-ET-1 levels were identified in COPD patients with PAH, and significant correlation was detected between EBC-ET-1 levels and Pulmonary Artery Systolic Pressure (PASP) values.

Mansoor et al. [209] applied EBC proteomic analysis using GM/MS in order to detect volatile compounds (VOCs), which revealed a unique EBC-metabolic fingerprint for IPAH patients. 32 VOCs were detectable only in patients with IPAH, and 6 of them were significantly associated with haemodynamic variables of the pulmonary circulation.

#### 3.5.4. Sarcoidosis

Piotrowski et al. [9] applied comparative analysis of EBC and BAL in patients with sarcoidosis, which detected high levels of 8-isoprostane and Cys-LTs in both biological materials, as well as significant correlation among their levels.

In a study by Ahmadzai et al. [210], significantly higher levels of EBC-neopterin and TGF-*β*1 were identified in patients with sarcoidosis compared to healthy controls, whereas no significant difference in levels of EBC-angiotensin converting enzyme was detected between the two populations.

#### 3.5.5. Obstructive Sleep Apnea Syndrome (OSA)

Studies in pediatric populations with OSA (clinical score > 40) detected significantly increased EBC-IL-6 and 8-isoprostane concentrations compared to healthy controls. The abovementioned biomarkers were significantly correlated with clinical score and cardiac dysfunction [211]. Additionally, increased levels of EBC-LTB_4_ and Cys-LTs were found significantly increased in children with Apnea-Hypopnea Index (AHI) > 5/h and they were associated with severity of the disease [212]. Furthermore, elevated morning-EBC-H_2_O_2_ levels were found in pediatric population with severe OSA. This finding is indicative of the intense, night long oxidative stress [213], which is also expressed by the significant increase of EBC-uric salts [214].

Studies enrolling adult population with OSA have also detected a significant raise of EBC markers of oxidative stress. More specifically, significantly increased levels of 8-isoprostane [215–217], TNF-*α*, IL-6 [216, 217], H_2_O_2_ [218], and reduced pH [217] have been identified compared to both healthy and obese controls. EBC-8-isoprostane concentration has been found significantly higher immediately after morning awakening and it has been correlated to AHI score and neck periphery. Furthermore a significant reduction of EBC-8-isoprostane levels has been found after CPAP therapy [215]. In addition, significantly reduced EBC-H_2_O_2_ levels have been identified after 6 weeks of CPAP therapy (from 450 ± 130 nmol/L to 294 ± 110 nmol/L) [218].

It must be pointed out that EBC-ICAM-1 and IL-8 have been found equally increased both in healthy obese adults and in obese adults with OSA [219], implying that the abovementioned inflammatory markers are not OSA specific.

#### 3.5.6. Systemic Lulus Erythematosus (SLE)

Nielepkowicz-Goździńska et al. detected significantly increased IL-8 [220], IL-6, and IL-10 [221], both in EBC and in BAL of SLE patients. Furthermore, the abovementioned changes were significantly correlated with the disease activity [222].

#### 3.5.7. Chronic Renal Disease (CRD)

Significantly increased EBC-pH, nitrites, and nitrates have been demonstrated in patients with end stage CRD. Moreover, levels of the abovementioned biomarkers decreased after haemodialysis [222]. Additionally, 22-fold EBC-H_2_O_2_ concentration has been demonstrated in patients with severe uremia who have undergone haemodialysis compared to healthy controls [223].

#### 3.5.8. Pharmacokinetics

EBC proteomic analysis has also been applied in toxicology, for detection of several compounds, including drugs. More specifically, EBC analysis through mass spectrometry can potentially be used for detection of drug metabolites concentrations during patient follow-up [224]. A well-studied example is the detection of EBC-methadone through LC-TMS analysis [225] (Table 5 refers to EBC biomarkers detected in all the above mentioned respiratory and systemic diseases).

## 4. Discussion and Conclusion

This study is a systematic review of the literature, presenting both the theoretical principles and the potential clinical applications and limitations of the contemporary EBC analysis.

Bibliographic investigation detected a prominent research activity on that field, resulting in rapidly increasing number of innovative papers published every day, concerning almost all the areas of modern respiratory medicine.

Study of exhaled air has been an area that has garnered interest since the beginning of medical science and investigation. From the Hippocratic medicine (uremic breath) [226] to the modern applications of e-nose [227], the study of exhaled breath, aiming to reveal clinically useful pathogenetic mechanisms of various diseases, has undergone a remarkable evolution.

EBC collection and analysis are the most complete product of that long-term research. It is undoubtedly an advantageous technique, when noninvasive, effortless, and painless collection of respiratory biological material is concerned. Furthermore, it is innovative, since EBC is proved to be a well manageable matrix for application of all the technologically advanced methods of contemporary biomedical research. In this way, EBC-analysis produces findings expressing a considerable part of proteomic, metabolic, and genomic fingerprint.

However, in order for a new technique to be introduced in clinical practice, high quality of standardization is required, both in sampling and in interpretation. In regard to sampling and EBC collection procedures, there are reliable guidelines [1], which have been applied in the vast majority of the original research studies mentioned in the present review, but they already count 10 years from their publication (2005). Therefore, they lack updated knowledge coming from proteomic and genomic analysis, especially in lung cancer and systemic diseases. Many promising biomarkers have been detected, especially in the genomics area, creating new perspectives. Moreover, the guidelines focus on inflammatory and oxidative stress biomarkers detected in early pilot studies, the results of which are questioned, since they are contradictory with recent data, demonstrating that such biomarkers are nonspecific and with low diagnostic accuracy. Thus, recent studies should be added to an updated version of ATS/ERS task force dedicated to EBC.

In regard to standardization of EBC-analysis, it must be pointed out that there is absence of large cohorts towards determination of normal-reference values of EBC-biomarkers [228] and therefore normal proteomic, metabolic, and genomic “fingerprints” of exhaled breath have not been adequately characterized yet. Furthermore, existing knowledge is based on data provided by small scale studies, often demonstrating conflicting results, possibly attributable to the poor reproducibility of EBC biomarkers [137]. There is a methodological limitation of existing studies that predisposes to such poor repeatability. In the vast majority of studies, especially ones on asthma and COPD, the different inflammatory pathways are not distinguished and thereby inflammatory heterogeneity of enrolled patients could possibly be a factor associated with poor reproducibility. Patients with a neutrophilic pattern of inflammation, for example, will possibly have different EBC-mediators from patients with an eosinophilic or a paucigranular inflammatory pattern and a different response to treatment. Therefore, despite the intensive research on asthma, COPD, and lung cancer, there is still no specific marker useful for diagnosis, follow-up, response to treatment, and prediction of exacerbation.

There is also a lack of screening and prospective studies enrolling asymptomatic patients, in order to evaluate if EBC markers could identify a preclinical lung disease condition. Such studies, demonstrating results of clinical significance, could potentially become the brink of a major breakthrough in respiratory medicine. Such results, combined with existing portable EBC collection equipment and the easy, noninvasive sampling, could make EBC an exceptionally promising screening method for early detection of lung disease.

Summarizing, it seems that EBC-analysis is an intriguing achievement of biomedical research, but still in early stage when it comes to its application in clinical practice. Current knowledge, however, creates optimism regarding the potential of EBC-analysis to reveal disease-specific fingerprints, as the sum of many mediators evaluated with new technologies, able to characterize a disease or its exacerbation.

## Figures and Tables

**Table 1:** Changes of exhaled breath condensate biomarkers in asthma.

Biomarkers	Stable asthma	Unstable asthma
pH	↔ [45, 71–73] ↓ [62–65] ↑ after ICS treatment [66, 69, 72] ↓ in GERD [63, 116, 117], ↑ after lansoprazole ↔ in GERD [118], ↔ after lansoprazole	↓↓ [62, 66–68]

H_2_O_2_	↑ [74] correlated with ↓ FEV_1_ ↑ [75] correlated with severity, ↓ FEV_1_, and ↓ after treatment with corticosteroids	

LTs	↑ [15, 76, 80–87]	
Cys-LTs	↑ in EIB, before exercise [78]	Cys-LTs ↓ after OCS treatment [89]
	↑ in EIB, 10 min after exercise [78]	
LTE_4_	↑ but ↔ after ICS treatment [82]	

8-Isoprostane	↑ but ↔ after ICS, OCS treatment [82, 93, 94]	

PGE_2_	Nonsmokers: ↔ [15, 82, 94] smokers: ↑ [95]	

ILs		
IL-4	↑ [96–101]	
IL-5	↑ [102]	
IL-6	↑ [97–100, 103]	
IL-8	↑ [104]	
IL-10	↑ [70]	
IL-17	↑ [104]	

INF-*γ*	↓ in paediatric population [96]	

RANTES	↑ [104, 106, 107] correlated with ↓ FEV_1_ and ↑ R_aw_ [104]	↑↑ [106, 107]

MIP1*α*, MIP1*β*	↑ in steroid naive adults [104]	

TNF-*α*, TGF-*β*	↑, correlated with nonspecific BHR(PC) [104]	

ET-1	↑ [106, 107]	↑↑ [106, 107]

Cytokeratine 1	↑ in paediatric population [109]	

MDA	↑, ↓ after ICS treatment [110]	

ADMA	↑ In paediatric population [111]	

CCL11	↑ [112]	↑↑ [112] ↓ after omalizumab therapy in severe asthmatic patients [113]

hs-CRP	↑ correlated with ↑ FeNO and ↑ serum hs-CRP [114]	↑↑ correlated with ↑ FeNO and ↑ serum hs-CRP [114]

sICAM-1	↑ related to bronchial asthma manifestation in paediatric population [115]	

↑ = increase, ↓ = reduction, ↔ = no significant change, and [*x*] = corresponding reference.

ICS = inhaled corticosteroids, GERD = gastroesophageal reflux, H_2_O_2_ = hydrogen peroxide, LTs = leukotrienes, PGE_2_ = prostaglandin-E_2_, EIB = exercise-induced bronchoconstriction, OCS = oral corticosteroids, ILs = interleukins, INF-*γ* = interferon-*γ*, MIP = macrophage inflammatory proteins, TNF-a = tumor necrosis factor-a, TGF-*β* = transforming growth factor-*β*, BHR(PC) = bronchial hyperresponsiveness(provocative concentration), ET-1 = endothelin-1, SPs = surfactant proteins, MDA = malondialdehyde, ADMA = asymmetric dimethylarginine, CCL11 = eotaxin-1, hs-CRP = high sensitivity c-reactive protein, and sICAM-1 = solute intercellular adhesion molecule-1.

**Table 2:** Short and long-term effects of tobacco smoking on exhaled breath condensate.

Biomarkers	Immediate effect	Long-term effect
pH	↔ in healthy smokers [121, 122]	↔ in healthy smokers with <10 py [122, 126] ↓ in healthy smokers with >10 py [122, 126]
	↑ in healthy smokers [41]	
	↓ in smokers with allergic rhinitis [123]	
	↓ in smokers with bronchial asthma [121, 124]	
	↓ after SHS in healthy nonsmokers [130]	

H_2_O_2_	↑ after SHS in healthy nonsmokers [130]	

8-Isoprostane	↔ [121]	
	↑ [46]	

ILs		
IL-4, 5, 10, 17	↓ after waterpipe smoking [125]	
IL-6		↑ [116, 127], correlated with FVC, FEV_1_, and eCO levels and smoking duration [127, 128]
IL-8	↔ [122]	↑ [127, 128]

TNF-*α*		↑, correlated with FVC and FEV_1_ values [129]

INF-*γ*	↓ after waterpipe smoking [125]	

Nitrates	↑ [45]	

LTB_4_		↑ [116], ↑↑ in smokers with allergic rhinitis [130]

EBC electric conductivity (*μ*S)	↑ [122]	

Haemoglobin *β*-chain		Detected [144]

Data represent changes after cigarette smoking. When changes refer to secondhand cigarette smoking or other tobacco product effects, there is a special reference inside the table.

↑ = increase, ↓ = reduction, ↔ = no significant change, and [*x*] = corresponding reference.

py = pack-years, SHS = secondhand cigarette smoking, H_2_O_2_ = hydrogen peroxide, ILs = interleukins, TNF-a = tumor necrosis factor-a, INF-*γ* = interferon-*γ*, and LTB_4_ = leukotriene-B_4_.

**Table 3:** Changes of EBC biomarkers in chronic obstructive pulmonary disease (COPD).

Biomarkers	Stable COPD	AECOPD
pH	↔ [132]	↓↓, and ↑ after treatment [145]
	↓ [57, 132, 134], correlated with ↓ FEV_1_ [57]	
	↓, correlated with GOLD stage, IC/TLC, RV/TLC, and CO diffusing capacity, only in ex-smokers [134]	

H_2_O_2_	↑ [135–137]	↑, and ↓ after treatment [146]

Aldehydes		
(MDA, hexanal, heptanal)	↑ [138, 139]	
	↔ [140]	

8-Isoprostane	↑ [136, 141, 142]	↑, and ↓ after treatment [146]
	High variability within day and between days [143]	

LTs		
LTB_4_	↑ [16, 141, 152], correlated with ↓ CO diffusing capacity	↑ [146, 147], and ↓ after treatment [146]
		↑, and ↓ after recovery [152]
LTE_4_	Undetectable [16]	
	High variability within day and between days [143]	
Cys-LTs		↑ [3]

PGs		
PGE_2_, PGF_2_	↑ (compared to asthmatic patients) [16]	

Acute phase response		
AAT, MMP-9, TIMP-1		↑ [150, 151]
Neutrophilic inflammation		
IL-1*β*, IL-12	↑ [52]	
IL-1a, IL-1*β*,	Dominant protein type (62%) [144]	
IL-2, IL-12a,		IL-1*β*, IL-6,
IL-12*β*, IL-15,		IL-8, IL-10, ↑↑[52, 148, 149]
INF-*α*, INF-*γ*,		IL-12, TNF-a
TNF-a, C3		
NCA, GRO-a		↑, and ↓ after treatment (only in outpatients) [152]

Nitrites		↑ [149]

*α*_1_-antitrypsin, SP-A_1_, SP-A_2_, S-100 calgranulins	Detected in COPD, but not in A_1_AD patients [144]	

IL-1*α*, lysozyme-C	Detected in A_1_AD, but not in the other COPD patients [144]	

sHLA-1, sCD95, SLP-1, MPO		↑ [153, 154]

Nucleic acids of		
influenza virus, RSV, *L. pneumophila *		Detected [155]

↑ = increase, ↓ = reduction, ↔ = no significant change, and [*x*] = corresponding reference.

AECOPD = acute exacerbation of COPD, FEV_1_ = forced expiratory volume at 1 sec, IC = inspiratory capacity, TLC = total lung capacity, RV = residual volume, CO = carbon monoxide, MDA = malondialdehyde, H_2_O_2_ = hydrogen peroxide, LTs = leukotrienes, PGs = prostaglandins, ILs = interleukins, INF = interferon, TNF-a = tumour necrosis factor-a, C3 = complement fraction 3, NCA = neutrophil chemotactic activity, AAT = a1-antitrypsin antiprotease, MMP-9 = metalloproteinase-9, TIMP-1 = metalloproteinase-1 inhibitor, SPs = surfactant proteins, s = solute, SLP-1 = secretory leukocyte protease inhibitor-1, MPO = myeloperoxidase, RSV = respiratory syncytial virus, and *L. pneumophila* = *Legionella pneumophila*.

**Table 4:** Exhaled breath condensate biomarkers in lung cancer.

Biomarkers	Lung Cancer	
H_2_O_2_	↑, correlated with ↑ urine-MDA [156]	

Inflammatory markers		
ILs		
IL-2	↑ [158]	NSCLC
IL-6	↑ [157, 169]	
IL-17	↑ [169]	
TNF-*α*	↑ [158, 159, 161]	
IL-1*β*, IL-6	↑ after lobectomy or pneumonectomy [164]	
IL-8, TNF-a, sICAM-1		

Leptin	↑ in NSCLC [158]	

ET-1	↑ in NSCLC [160]	

VEGF	↑ [159, 161, 165], correlated with ↑ IL-6, IL-17, TNF-*α*, and with tumor diameter in CT [159]	NSCLC
	↓ after chemotherapy [165]	

bFGF	↑ in NSCLC and ↓ after chemotherapy [165]	

COX-2	↑ in NSCLC patients along with the disease stage [160]	
survivin		

MMP-9	↑ in NSCLC patients along with the disease stage [158]	

GRTP-1	Detected in a single NSCLC patient [159]	

CCL28	↑ in lung cancer [162] ↔ no difference in cytokine signals [163]	
MIP-3		
GRo-a		

Eotaxin-2	↓ in lung cancer [162] ↔ no difference in cytokine signals [163]	
Eotaxin-3		
FGF-6		
FGF-7		
IL-10		

Tumor markers		
CEA	↑ in NSCLC and strongly correlated with histological type [169]	
SCC		
NSE		

Genomic analysis		
Somatic mutations of p53	Detected [170]	
Microsatelite DNA instability and loss of heterozygosity: 3p chromosome (locus D3S2338, D3S1266, D3S1304, D3S1289)	Detected in NSCLC patients [171]	
loss of heterozygosity: 3p chromosome (locus D3S1289)	Correlated with negative prognostic value in adenocarcinoma and poor prognosis in NSCLC [172]	
Microsatelite DNA instability and loss of heterozygosity: 3p chromosome (locus D3S2338)	Correlated with poor survival among patients with squamous cell lung carcinoma [173]	
ERCC1 and ERCC2 genes (chromosome 19q)	Increased risk of NSCLC among smokers [174]	
miRNA	↑ miRNA-21 with ↓↓ miRNA-486: proposed as screening test for NSCLC diagnosis [175]	
KRAS mutation in codon 12	↓ after NSCLC resection surgery [176]	
Methylation of DAPK, PAX5*β*, and p16 genes	Detected in patients with NSCLC [6, 177]	
HPV genome	Detected in patients with NSCLC [178]	

↑ = increase, ↓ = reduction, ↔ = no significant change, and [*x*] = corresponding reference.

H_2_O_2_ = hydrogen peroxide, MDA = malondialdehyde, NSCLC = non-small cell lung cancer, ILs = interleukins, TNF-a = tumour necrosis factor-a, ET-1 = endothelin-1, VEGF = vascular endothelial growth factor, bFGF = basic fibroblast growth factor, COX-2 = cyclooxygenase-2, MMP-9 = metalloproteinase-9, GRTP-1 = growth hormone regulated TBC protein-1, CCL-28 = mucosae-associated epithelial chemokine (MEC), MIP = macrophage inflammatory proteins, GRO-a = growth related oncogene-a, FGF-6 = fibroblast growth factor-6, FGF-7 = fibroblast growth factor-7, CEA = carcinoembryonic antigen, SCC = squamous cell carcinoma antigen, NSE = neuron-specific enolase, miRNA = micro-RNA, and HPV = human papillomavirus.

**Table 5:** Exhaled breath condensate biomarkers in several lung and systemic diseases.

Disease	Biomarkers	Changes
Idiopathic pulmonary fibrosis (IPF)	8-Isoprostane H_2_O_2_ 3-nitrotyrosine NO_*x*_ docosatetraenoyl-LPA	↑ [194, 195]

Cystic fibrosis (CF)	pH	↓, and ↓↓ during exacerbation [196, 201], ↔ [197]
	Nitrotyrosine	↑ [198], ↔ 3-nitrotyrosine [199]
	Nitrites	↑ [200, 201]
	Nitric oxide	↓, correlated with disease severity [201]
	8-Isoprostane	↑, marker of CF exacerbation [201]
	IL-6	↑ during exacerbation, and ↓ after treatment [203, 204]
	IL-8	↔ in stable CF [202], ↑ in CF exacerbation, and ↓ after treatment [203, 204]
	IL-5 TNF-*α*	Detected in paediatric CF population [208]
	LTE_4_	↑ during exacerbation, and ↓ after treatment [203, 204]

Pulmonary arterial hypertension (PAH)	Natriuretic peptide pro-BNP ET-1	Higher levels in IPAH versus COPD-PAH [206]
	ET-1	↑ in COPD-PAH, correlated with PASP [208]
	6-keto-PGF_1*α*_ 8-isoprostane IL-6	Higher levels in COPD-PAH versus IPAH [207]

Sarcoidosis	8-Isoprostane Cys-LTs	↑ in both EBC and BAL [9]
	Neopterin TGF-*β*1	↑ [210]

Obstructive Sleep Apnea Syndrome (OSA)		
Pediatric patients	8-Isoprostane IL-6	↑ in patients with clinical score >40, correlated with cardiac dysfunction [211]
	LTB_4_ Cys-LTs	↑ in patients with AHI >5/h [212]
	H_2_O_2_	↑ [213]
	Uric salts	↑ [214]
Adult patients	8-Isoprostane	↑ [215–217], correlated with AHI and neck periphery, and ↓ after CPAP therapy [215]
	IL-6	↑ [216, 217]
	TNF-*α*	↑ [216, 217]
	pH	↓ [217]
	H_2_O_2_	↑, and ↓ after after CPAP therapy [218]
	ICAM-1 IL-8	↑ in both healthy obese and obese OSA-patients [219]

Systemic Lupus Erythematosus (SLE)	IL-6, IL-8, IL-10	↑ in both EBC and BAL [220, 221], significantly correlated with the disease activity

Chronic Renal Disease (CRD)	pH Nitrites Nitrates	↑, and ↓ after haemodialysis [222]
	H_2_O_2_	↑ in patients with severe uremia, who underwent haemodialysis [223]

↑ = increase, ↓ = reduction, ↔ = no significant change, and [*x*] = corresponding reference.

H_2_O_2_ = hydrogen peroxide, NO_*x*_ = nitric oxides, ILs = interleukins, TNF-a = tumor necrosis factor-a, LTE_4_ = leukotriene-E4, pro-BNP = pro-brain natriuretic peptide, ET-1 = endothelin-1, LTs = leukotrienes, TGF-*β*1 = transforming growth factor-*β*1, LTB_4_ = leukotriene-B_4_, and ICAM-1 = intercellular adhesion molecule-1.
